# Real-World Surgical Experience with Thoracic Solitary Fibrous Tumors: Outcomes from a High-Volume Indian Center

**DOI:** 10.1007/s13193-025-02349-x

**Published:** 2025-05-30

**Authors:** Vishnu S. Menon, Sabita Jiwnani, Karthik Venkataramani, Virendra Tiwari, Devayani Niyogi, Kunal Gala, Rutvij Khedkar, Rajiv K. Kaushal, Pramesh CS

**Affiliations:** 1 Department of Surgical Oncology, Tata Memorial Hospital, Homi Bhabha National Institute, Mumbai, 400012 Maharashtra India; 2 Division of Interventional Radiology, Department of Radiodiagnosis, Tata Memorial Hospital, Homi Bhabha National Institute, Mumbai, 400012 Maharashtra India; 3 Department of Pathology, Tata Memorial Hospital, Homi Bhabha National Institute, Parel, Mumbai, Maharashtra India; 4 Thoracic Surgical Services, Department of Surgical Oncology, Tata Memorial Hospital, Homi Bhabha National Institute, Mumbai, 400012 Maharashtra India

**Keywords:** Sarcoma, Pleura, SFT, Angioembolization, Thoracic masses

## Abstract

This study evaluates the surgical approach, role of trans-arterial angioembolization (TAE) and long-term outcomes of patients presenting with solitary fibrous tumors (SFT) to a tertiary thoracic surgical unit in India. This is a retrospective study of thoracic SFTs operated between 1 August 2008 and 31 March 2024 from our center. The clinical, radiological, and histo-pathological features, surgical outcomes, and follow-up status were retrieved from the surgical database and electronic medical records and evaluated. A total of 34 patients underwent surgical excision during the abovementioned period. The median tumor volume calculated from preoperative computed tomography images was 765.8 cm^3^ (range from 13 to 3740 cm^3^). Preoperative TAE was employed in 15 patients, all of whom had tumors larger than 10 cm in the largest dimension. Complete microscopic tumor (R0) resection was achieved in all cases. Major surgical morbidity with Clavien-Dindo grade 3 or more was seen in 11.8% (4/34) and perioperative mortality was 5.8% (2/34). Four recurrences were observed, with a disease-free interval ranging from 13 to 73 months, and no local recurrences were observed. All failures were systemic failures in the lung and had dismal outcomes despite salvage treatment. The 5-year disease-free survival and overall survival were 77% and 82%, respectively. Surgical extirpation with negative margins is the cornerstone of treatment for thoracic SFTs. Preoperative TAE is useful in the management of SFTs of 10 cm or more in size. Recurrences are late systemic failures occurring in non-low risk Demicco group patients and have poor outcomes.

**Graphical Abstract:**

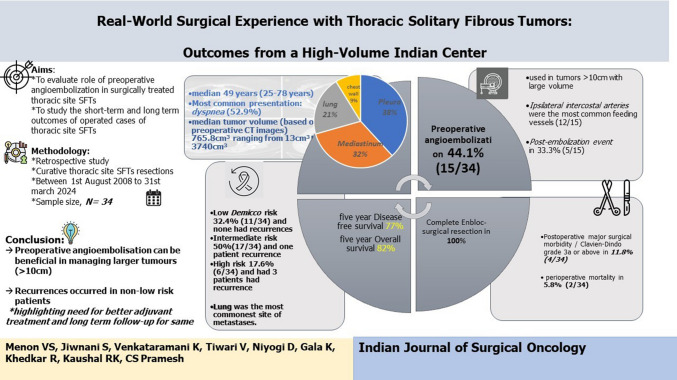

## Introduction

Solitary fibrous tumors (SFTs) are rare mesenchymal tumors most commonly arising in pleural spaces [1, 2]. These tumors have an annual incidence of 2.8 persons per one lakh persons per year [1, 2]. They are among the five commonest pathologies presenting as giant thoracic tumors (GTT). GTTs are defined as thoracic masses of 10 cm or more on the long axis or that occupy the whole hemithorax [3, 4]. Surgical resection is the treatment of choice, but challenges to en bloc resection include their relatively larger sizes at presentation, increased vascularity, and close proximity to vital structures within the thorax [2, 5, 6]. They can reach very large proportions, growing by displacing rather than invading adjacent structures, and can present with a wide array of symptoms [7]. SFTs are also known to be associated with paraneoplastic events like hypertrophic pulmonary osteoarthropathy and reactive hypoglycemia [8]. Due to their slow-growing and vascular nature, they develop multiple feeders and collateral vessels, arterial as well as venous from surrounding vessels, leading to major blood loss during surgery [3, 7]. The role of angioembolization in such large vascular thoracic masses is described and remains a useful tool to possibly reduce intraoperative blood loss, potentially yielding en bloc resection and easy handling of tissue [9]. While the spectrum of presentation and management is wide ranging, their biological behavior also represents a spectrum, ranging from stable, benign small-sized long-standing masses to rapidly enlarging malignant tumors occupying the entire hemi-thoracic cavity. Though the terms *Benign* and *Malignant* SFTs are no longer used, several attempts have been made to develop more comprehensive risk stratification models which could better predict the biological potential of these tumors [10–12]. The Demicco risk model, which is one of the most studied models in this regard, is a three-tier model that takes into account both the age of the patient and pathological features [11]. Recurrences have been variably described, ranging from 5 to 45%, and are mostly systemic, with the lung being the commonest site of failure [13]. However, due to the rarity of this disease and lack of published literature in this respect from our region, there is a general lack of consensus, and most treatment options are based on anecdotal evidence. The aim of this study was to explore the spectrum of surgically treated thoracic site SFTs, the role of preoperative trans-arterial angioembolization (TAE), short-term surgical outcomes, and long-term oncological outcomes including their pattern of recurrences.

## Methods

### Study Design, Population, and Exclusion Criteria

This study was a retrospective cohort analysis conducted at the thoracic surgical unit of a tertiary cancer center in India. A prospectively maintained institutional database from the electronic medical records (EMR), between 1 August 2008 and 31 March 2024, was assessed to identify patients operated on for thoracic site SFTs, which included intrathoracic as well as chest wall SFTs. The WHO histological classification criteria, which were updated in 2020, were used as the diagnostic criteria for SFT [12]. The essential criterion was the presence of a well-circumscribed mass with spindle to ovoid cells arranged around a branching and hyalinized vasculature; variable stromal collagen deposition; and CD34 and/or STAT6 expression by immunohistochemistry. The desirable criterion was the demonstration of *NAB2*-*STAT6* gene fusion. The following inclusion criteria were applied: (1) biopsy-proven SFT meeting the essential and/or desirable diagnostic criteria, (2) epicenter of disease in thoracic sites including the mediastinum, lung, pleura, or chest wall, (3) underwent curative resection from our center during the said duration. The following exclusion criteria were applied: (1) extra-thoracic site SFT, (2) recurrent disease, (3) less than 6 months duration of follow-up. The study adhered to ethical standards set by the institutional research committee and the Indian Council of Medical Research, in alignment with the principles in the 1964 Helsinki Declaration and subsequent amendments [14]. This study was performed in accordance with the Strengthening the Reporting of Observational Studies in Epidemiology (STROBE) guidelines [15].

### Preoperative Evaluation of Patients, Tumor Volumetry, and Risk Assessment

The decisions regarding diagnosis, need for preoperative biopsy, operability, and approach to the surgery were made in a multi-disciplinary tumor board. Biopsy and surgical specimens were reviewed and reported by pathologists trained in thoracic pathology. The extent of the disease was confirmed with preoperative imaging, operative notes, and histopathology reports. Tumor volume was calculated by retrieving images from the Picture Archiving and Communication System (PACS) of our center, and tumor dimensions were calculated in centimeters, including height (***a***), anteroposterior length (***b***), and medio-lateral width (***c***) in its largest dimension. Tumor volume was calculated as the product of 0.5 × ***a*** × ***b*** × ***c*** [16]. Risk assessment was done based on the Revised Demicco model and the de Perrot staging. The Demicco risk stratification includes four parameters, which are age, size of the tumor, mitosis rate, and necrosis percentage, and has three tiers (low, intermediate, and high) [11, 17]. The de Perrot staging used the absence/presence of pleural pedunculation with histologic features of malignancy and the presence of metastasis to create a five-tiered staging system [18].

### Data Retrieval and Defining End-points

Clinical information including patient demographics, pathological characteristics, surgical details, postoperative outcomes, and oncological outcomes of patients operated for SFTs were collected from the database and EMR. All patients were followed up until 30 September 2024 (minimum 6 months post surgery) and the duration of follow-up was calculated from the date of surgery to the last date of follow-up as recorded in the EMR. Overall survival (OS) was calculated as the duration in months from the date of surgery to the date of death due to any cause or if surviving to the last follow-up. Disease-free survival (DFS) was calculated as the duration in months from the date of surgery to the date of local or metastatic recurrence or death due to any cause or to the last follow-up.

The primary end points of this study were to evaluate the pattern of presentation of thoracic site SFTs, the role of preoperative TAE, the surgical approach, short-term surgical outcomes, and the pattern of recurrences. The secondary end points of this study were to evaluate the OS and DFS of the cohort and the potential impact of various factors on OS and DFS, respectively.

### Statistical Analysis

Data was analyzed with IBM SPSS version 29 [19]. Descriptive statistics were used to analyze clinical, pathological, and radiological features and management protocols. Categorical variables were presented as numbers with percentages and were compared among groups using chi-square *t* test and Fisher’s exact test when needed. Distributions of continuous variables were reported as median with range and were compared using the Mann–Whitney* U* test. Outcomes, complications, OS, and DFS were analyzed using nonparametric calculations. Kaplan–Meier survival curves were used to estimate survival, and the log-rank test was used to assess differences between defined groups. We measured the hazard ratios (HR) with the 95% confidence intervals (CI) and carried out a multivariate analysis for OS and DFS using the Cox proportional hazards (CPH) model. We took a *p* value of 0.05 or less as significant.

## Results

### Cohort Characteristics (Table 1)

**Table 1:** Demographics, clinicopathologic features, treatment characteristics, and outcomes of the study cohort

Parameter	Median (range)		Mean ± standard deviation (SD)
1. Age	49 years (25–78 years)		49.5 +/–11.7 years
	Total number *N* (%)	Recurrence *N*_1_ (%)	OS events *N*_2_ (%)
2. Sex			
*Male*	19 (55.9)	3(15.7)	4 (21.1)
*Females*	15(44.1)	1(6.6)	2(13.3)
3. Sites			
*Mediastinum*	11 (32.4)	2 (18)	3 (27.2)
*Parietal pleura*	13 (38)	1 (7.6)	2 (15.3)
*Visceral pleura/lung*	7 (20.6)	1 (14.2)	1 (14.2)
*Chest wall*	3 (8.8)	0 (0)	0 (0)
4. Size of tumor			
*0 to 5 cm*	1 (2.9)	0 (0)	0 (0)
*5 to 10 cm*	7 (20.5)	0 (0)	0 (0)
*10 to 15 cm (GTT)*	12 (35.3)	2 (16.7)	3 (25)
*More than 15 cm (GTT)*	14 (41.2)	2 (14.2)	3 (21.4)
5. Symptoms			
*Breathlessness*	18 (52.9)		
*Chest pain*	8(23.5)		
*Chronic cough*	1 (2.9)		
*Chest wall swelling*	3 (8.8)		
*Incidental*	1 (2.9)		
*Hypo-glycemia *^*(8)*^	1 (2.9)		
6. ASA grade			
*ASA1/ASA2*	27 (79.4)	4 (14.8)	4 (14.8)
*ASA3 or above*	7 (20.6)	0 (0)	2 (28.6)
7. Preoperative angioembolization			
*Yes*	15(44.1)	1 (6.6)	3 (20)
*Polyvinyl alcohol*	9 (26.4)		
*GelFoam*	1 (2.9)		
*Metal coils*	5(14.7)		
*No*	19(55.9)	3 (15.7)	3 (15.7)
8. Surgical approach			
*Sternotomy*	11 (32.4)		
*Thoracotomy*	15(44.1)		
*Clamshell*	3 (8.8)		
*Thoraco-abdominal*	1 (2.9)		
*Dartevell's approach*	2 (5.9)		
*Video-assisted thoracoscopic surgery*	2 (5.9)		
9. Lung resection			
*No*	10 (29.4)	1 (10)	1 (10)
*Yes*	24 (70.6)	3 (12.5)	5 (20.8)
*Wedge*	15(44.1)	0 (0)	2 (13.3)
*Lobectomy*	6(17.6)	2 (33.3)	2 (33.3)
*Pneumonectomy*	3 (8.8)	1 (33.3)	1 (33.3)
10. Mitotic count *(1/10 high power fields)*			
*0*	6 (17.6)		
*1–3*	20 (58.8)		
*4 or more*	8 (23.5)		
11. Tumor necrosis			
*No necrosis*	16 (47.1)		
*Less than 10%*	10 (29.4)		
*10% or more*	8 (23.5)		
12. de Perrot staging [18]			
*Stage 0*	7 (20.6)	0 (0)	0 (0)
*Stage I*	18 (52.9)	0 (0)	1 (5.5)
*Stage II*	2 (5.9)	1 (50)	1 (50)
*Stage III*	6(17.6)	2 (33.3)	3 (50)
*Stage IV*	1 (2.9)	1 (100)	1 (100)
13. Demicco risk groups [17]			
*Low*	11 (32.4)	0 (0)	0 (0)
*Intermediate*	17 (50)	1 (5.8)	2 (11.7)
*High*	6 (17.6)	3 (50)	4 (66.6)
14. England’s criteria [10]			
*Benign*	16 (47.1)	0 (0)	0 (0)
*Malignant*	18 (52.9)	4 (22.2)	6 (33.3)

*GTT* giant thoracic tumors, *ASA* American Society of Anaesthesiology (ASA) grading of preoperative health status, *cm* centimeter, *OS* overall survival

**Fig. 1:**
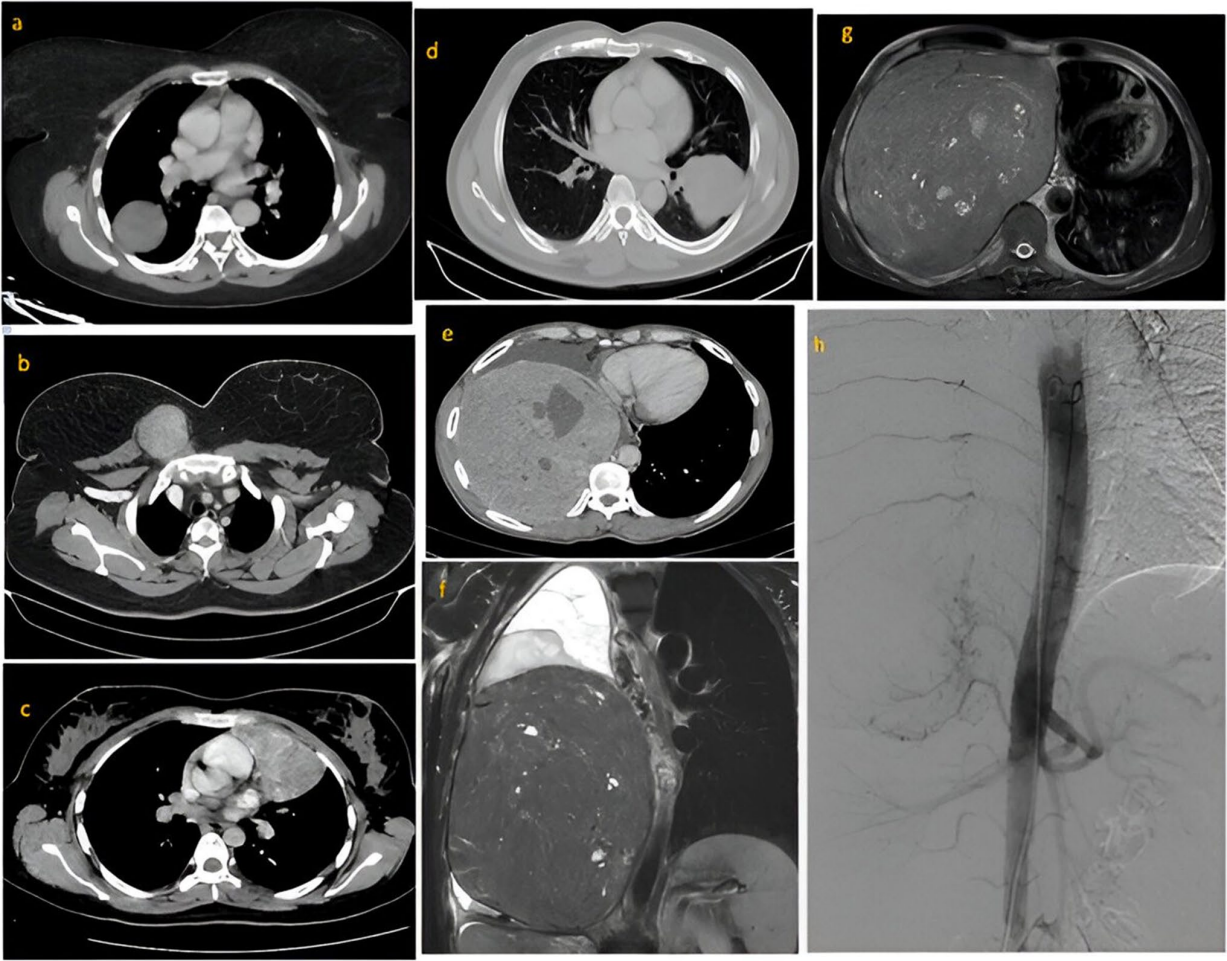
Radiological images showing different subsites of thoracic SFTs and a case of SFT presenting as a giant thoracic tumor. **a** Axial view of contrast-enhanced computed tomography (CT) images of right parietal pleural SFT in the right hemithorax. **b** Axial view of contrast-enhanced CT of right-sided anterior chest wall SFT. **c** Axial view of contrast-enhanced CT of left-sided anterior mediastinal SFT. **d** Axial view of contrast-enhanced CT of left lower lobe lung SFT. **e** Axial view of contrast-enhanced CT of thorax of a patient with giant thoracic tumor, which had a tumor volume of 2040 cm^3^. **f**, **g** Short T1 inversion recovery (STIR) coronal and axial magnetic resonance imaging (MRI) sequences of the thorax of the same patient. **h** Digital subtraction angiography images of the same patient showing feeders from the ipsilateral intercostal arteries and inferior phrenic artery, which were embolized

We identified 179 patients with SFT registered at our center for the said duration, of which 68 patients were thoracic site SFTs. Out of this, 34 patients who had undergone surgical resection formed the study cohort, and their median age at presentation was 49 years (range 25–78 years). The majority were males, with 19 patients (55.9%) and 15 were females (44.1%). Dyspnea was the most common presenting complaint, as observed in 18 patients (52.9%). We observed reactive hypoglycemia in one patient, which resolved after surgery. Contrast-enhanced computed tomography (CT) of the thorax was done in all patients at baseline, which was suggestive of an avidly enhancing mass with variable degrees of necrosis suggestive of a vascular tumor (Fig. 1). Parietal pleura was the likely epicenter, as seen in 13 patients (38.2%), followed by mediastinum in 11 (32.4%), lung in seven (20.6%), and chest wall in three (8.8%). Preoperative biopsy was done in all patients to establish the diagnosis. NAB2-STAT6 fusion testing, though the gold standard, was not performed in any patients, and all cases were diagnosed by their characteristic appearance and CD34 and/or STAT6 expression [12]. The median tumor volume calculated from preoperative CT images was 765.8 cm^3^, ranging from 13 to 3740 cm^3^.

### Pre-operative Angioembolization (Table 2, Fig. 1)

**Table 2:** Comparison between angioembolized and non-angioembolized SFTs

Parameters		Overall		Angioembolized		Non-angioembolized		*p* value
		Frequency (percentage)		Frequency (percentage)		Frequency (percentage)		
Overall		34		15		19		
Epicenter	*Parietal pleura*	13 (38.2)		9 (60)		4 (21)		0.008
	*Chest wall*	3 (8.9)		0 (0)		3 (10.9)		
	*Lung/visceral pleura*	7 (20.5)		0 (0)		7 (36.8)		
	*Mediastinum*	11 (32.4)		6 (40)		5 (26.3)		
Size	*0 to 5 cm*	1 (2.9)		0 (0)		1 (5.3)		0.003
	*5 to 10 cm*	7 (20.5)		0 (0)		7 (36.8)		
	*10 to 15 cm*	12 (35.3)		4 (26.6)		8 (42.1)		
	*More than 15 cm*	14 (41.2)		11 (73.3)		3 (15.7)		
Perioperative complications	*Clavien-Dindo (CD) Grade 3a or above*	4 (11.7)		3 (20)		1 (5.3)		0.113
	*Mortality*	2 (5.8)		2 (13.3)		0 (0)		0.2
Lung resection	Yes	24 (70.5)		10 (66.67)		14 (73.7)		0.656
Perioperative blood transfusion	Yes	20 (58.8)		12 (80)		8 (42.1)		0.026
		*Median (range)*	*Mean* ± *SD*	*Median (range)*	*Mean* ± *SD*	*Median (range)*	*Mean* ± *SD*	
Median blood loss (in mL)		650 (50–17,000)	1769 ± 3288	1400 (300–17,000)	2853 ± 4365	350 (50–8000)	913 ± 1801	0.057
Tumor volume (in cm^3^)		765.8 (13.5–3740)	901 ± 861.5	1240 (275–3740)	1478 ± 910	240 (13–1610)	446 ± 468	0.049
Length of hospital stay (LOHS, in days)		4 (3–60)	7.15 ± 9.93	5 (3–14)	6.93 ± 3.77	4 (3–60)	7.32 ± 13	0.3

*cm* centimeter, *SD* standard deviation

In 15 out of 34 patients (44.1%), preoperative angioembolization was employed and the median tumor volume of these cases was 1237.50 cm^3^ ranging from 275 to 3740 cm^3^. During angiography, feeding vessels were identified with a single feeder observed in four out of 15 patients, two feeders in six out of 15 patients, three feeders in three out of 15 patients, and more than three feeders in two out of 15 patients. The ipsilateral intercostal arteries were the most common feeding vessels embolized, as seen in 12 out of 15 patients, followed by the internal mammary artery in 10 cases and the inferior phrenic artery in five cases (Fig. 1). Successful angioembolization with confirmation of loss of blush was achieved in 100% of cases. Polyvinyl alcohol particles were the most commonly employed material for embolization in nine out of 15 cases, of which a combination with gel-foam particles was used in three cases. We observed intralesional broncho-pulmonary shunts in five cases (33.3%) which were managed with metallic micro-coil embolization. Post-embolization events in the form of transient fever, chest pain, and leukocytosis within 48 h were observed in five out of 15 (33.3%) patients. A major complication in the form of lower limb weakness was observed in one patient (6.6%), which was managed conservatively with later full regain of power. While in 10 out of 15 (66.6%) patients, surgery was done within 1 day after angioembolization; the maximum time from angioembolization to surgery in the cohort was 16 days, as observed in one patient.

### Surgical Approach and Postoperative Course

Surgical approach was planned based on tumor epicenter, with thoracotomy employed in 15 out of 34 cases. Midline-sternotomy was used in 11 cases, of which seven were mediastinal SFTs. Clam-shell approach was used in three out of 34 cases, of which two were pleural site SFTs. Dartevelle’s approach was utilized in two cases, both of which were pleural site SFTs. A combined thoraco-abdominal approach was required in one patient with diaphragmatic pleural SFT. Minimally invasive video-assisted thoracoscopic resections were achievable in 2 patients, both of which were lung SFTs. In all cases, en-bloc R0-resections were achievable. Lung resections were required in 24 cases, of which seven patients required the same for primary lung involvement and 14 patients required the same for intraoperative adjacent lung adherence or involvement. Additionally, three patients required lung excision due to inadequate lung expansion after removal of the mass, likely due to chronic collapse. Non-anatomical wedge resections were the commonest type of lung resection performed in 15 cases, followed by lobectomy in six cases and pneumonectomy in three cases. Among the three patients who underwent pneumonectomy, two were GTT SFTs with tumor volume of 1248 cm^3^ and 1512 cm^3^ with extensive lung parenchymal involvement; and the other reminder patient had extensive pulmonary hilar vascular involvement, though with a smaller tumor volume of 364 cm^3^. Chest wall resection was required in ten cases, with three cases requiring rib resections and reconstruction. One patient with posterior mediastinal SFT infiltrating into the 6 th thoracic vertebra required laminectomy and spine stabilization. Another patient who was metastatic on presentation with a single pancreatic metastasis underwent a second-stage resection of the pancreatic lesion after recovery from the thoracic procedure.

Median duration of hospital stay was 4 days (ranging from 3 to 50 days). Postoperative major surgical morbidity in the form of Clavien-Dindo grade 3a or above was 11.8% (4/34) and in perioperative mortality was 5.8% (2/34). Postoperative mortality was seen in patients with both large tumors (> 10 cm) and high surgical risk (American Society of Anesthesiology preoperative risk grade III) and was due to myocardial infarction and septic cardiomyopathy, respectively.

### Pathological Characteristics and Adjuvant Treatment (Table 1, Fig. 2)

**Fig. 2:**
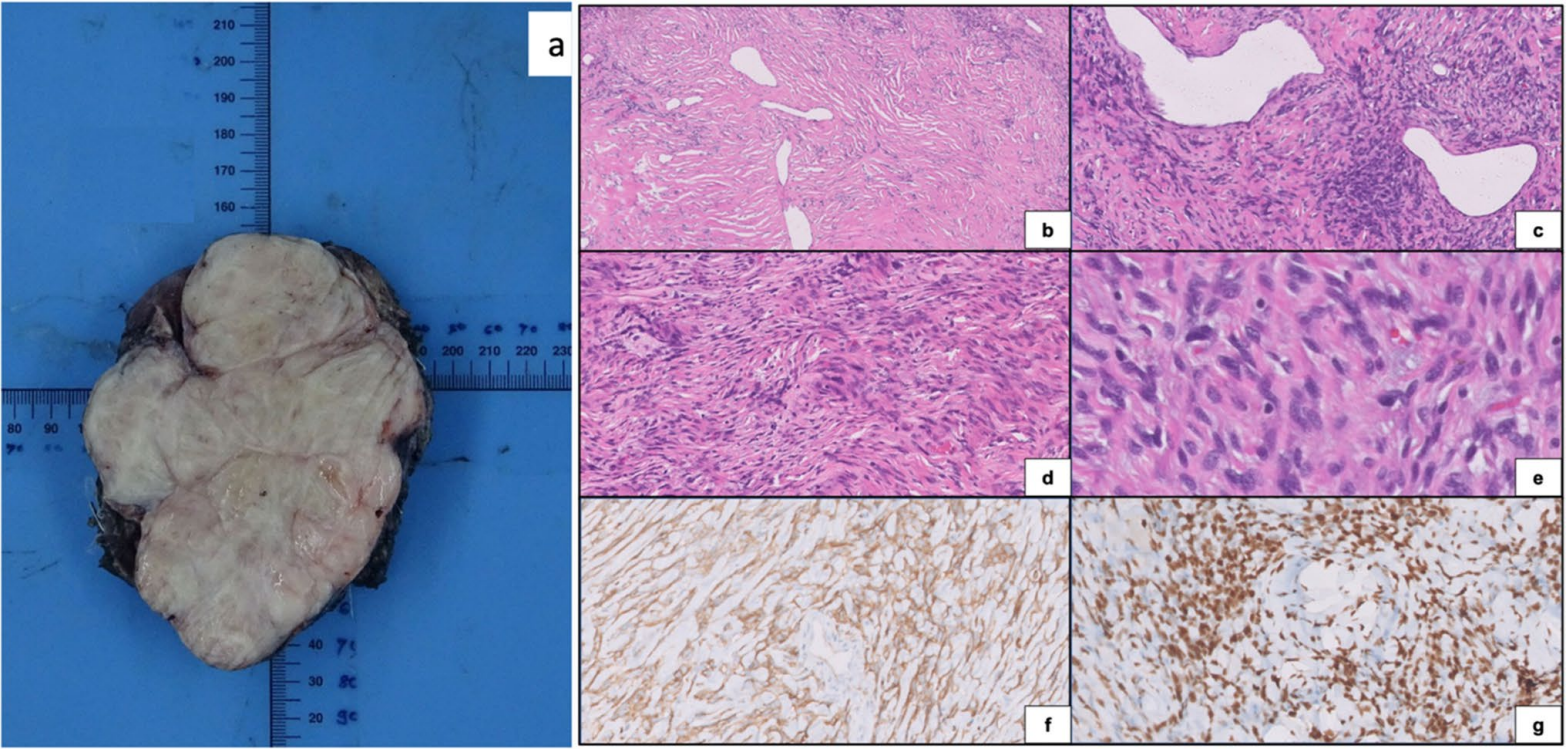
Gross image of a giant thoracic tumor arising from parietal pleura showing a well-circumscribed lobulated mass with gray-white surface and small area of cystic changes (**a**). Histologic features of the same case with prominent collagen-laying tumor cells and stag-horn-like vasculature (**b**, H&E at × 5), the tumor can show foci of variably increased cellularity (**c**, H&E at × 10), palisade-like arrangement in fibrous stroma (**d**, H&E at 10 ×), the tumor cells are bland, ovoid to spindled with no conspicuous mitoses (**e**, H&E at × 40), immunohistochemistry shows diffuse positivity for CD 34 (**f**, DAB at × 20), with strong diffuse nuclear staining for STAT6 (**g**, DABat × 20). Abbreviations: H&E, hematoxylin and eosin stain; CD34, cluster of differentiation 34; STAT6, signal transducer and activator of transcription 6; DAB, diaminobenzidine

A specific diagnosis of SFT was offered on preoperative biopsy in 26 cases (76.5%), and in the remaining eight patients (23.5%) reported as *spindle cell neoplasm* on preoperative biopsy, the diagnosis of SFT was established on the resection specimen. NAB2-STAT6 fusion testing was not done in any of the patients, but STAT6 IHC was positive in all 11 cases in which it was done. We observed CD34 as the commonest immunohistochemical marker used in our setting with 31 out of 32 cases testing positive [20]. Other markers BCL2 and S100 were 9 out of 12 cases and one out of seven cases, respectively, in which they were done. Despite 21 cases with primary involvement of lung and/or intraoperative suspicion of lung parenchymal involvement, only nine cases had lung parenchyma invasion in final histopathology, of which seven were initially classed as primary lung SFT. Microscopic negative margins were achieved in all patients. One patient who was oligometastatic on presentation, post surgery for primary and pancreatic metastases, received adjuvant chemotherapy. None of the patients in our cohort received radiotherapy. As per Demicco risk stratification model, out of the 34 patients, 11 patients (32.4%) were low risk, 17 patients (50%) were intermediate risk, and 6 patients (17.6%)were high risk. On bivariate Pearson correlation, Demicco risk score had a statistically significant correlation with CT-tumor volume (*p* = 0.003, Pearson correlation coefficient = 0.491).

### Survival Trends and Recurrence Patterns (Tables 3 and 4, Fig. 3)

**Table 3:** Univariate and multivariate analyses of disease-free survival (DFS)

Survival predictor		Disease-free survival (DFS)			
		Univariate		Multivariate	
		HR (95% CI)	*p* value	HR (95% CI)	*p* value
Age	60 years or less	1			
	> 60	1.305 (0.152–11.213	0.808		
Gender	Males	1.779 (0.325–9.745)	0.507		
	Females	1			
Site	Parietal pleura/chest wall	1			
	Lung/mediastinum	1.738 (0.318 to 9.501)	0.524		
Tumor volume	More than median volume (765.8 cm^3^)	1.143 (0.23 to 5.683)	0.871		
	Less than or equal to median volume (765.8 cm^3^)	1			
Size in largest dimension	10 cm or less	1			
	> 10 cm	1.832 (0.364–9.211)	0.463		
Preoperative angioembolization	No	1			
	Yes	1.817 (0.358–9.211)	0.471		
Lung resection	No	1			
	Yes	2.256 (0.261–19.505)	0.46		
Blood loss	Less than or equal to median loss (650 mL)	1			
	More than median loss (650 mL)	1.288 (0.258–6.422)	0.757		
Mitosis (per 10 high power field)	< 4	1			
	4 or more	1.476 (0.268–8.115)	0.655		
Necrosis	Absent	1			
	Present	77.3 (0.104–57723)	0.198		
Demicco risk grade [17]	Low/intermediate	1		1	
	High	10.225 (1.871–55.884)	0.007	4.130 (0.619–27.56)	0.143
de Perrot stages [18]		3.362 (1.325–8.532)	0.011	2.437 (0.885–6.714)	0.085

*HR* hazard ratios, *CI* confidence interval, *cm* centimeter, *mL* millilitre

**Table 4:** Univariate and multivariate analyses of overall survival (OS)

Survival predictor		Overall survival (OS)			
		Univariate		Multivariate	
		HR (95% CI)	*p* value	HR (95% CI)	*p* value
Age	60 years or less	1			
	> 60	1.195 (0.139–10.287)	0.871		
Gender	Males	1.682 (0.308–9.199)	0.548		
	Females	1			
Site	Parietal pleura/chest wall	1			
	Lung/mediastinum	1.920 (0.349 to 10.56)	0.453		
Tumor volume	More than median volume (765.8 cm^3^)	1.192 (0.240 to 5.927)	0.83		
	Less than or equal to median volume (765.8 cm^3^)	1			
Size in largest dimension	10 cm or less	1			
	> 10 cm	1.973 (0.393–9.910)	0.409		
Preoperative angioembolization	No	1			
	Yes	1.834 (0.364 to 9.250)	0.463		
Lung resection	No	1			
	Yes	2.310 (0.268 to 19.92)	0.446		
Blood loss	Less than or equal to median loss (650 mL)	1			
	More than median loss (650 mL)	1.324 (0.266 to 6.603)	0.732		
Mitosis (per 10 high power field)	< 4	1			
	4 or more	1.321 (0.240–7.260)	0.749		
Necrosis	Absent	1			
	Present	78.06 (0.104–58380)	0.197		
Demicco risk grade [17]	Low/intermediate	1		1	
	High	10.137 (1.851–55.526)	0.008	4.263 (0.650–27.936)	0.131
de Perrot stages [18]		3.403 (1.328–8.719)	0.011	2.497 (0.911–6.842	0.075

*HR* hazard ratios, *CI* confidence interval, *cm* centimeter, *mL* millilitre

**Fig. 3:**
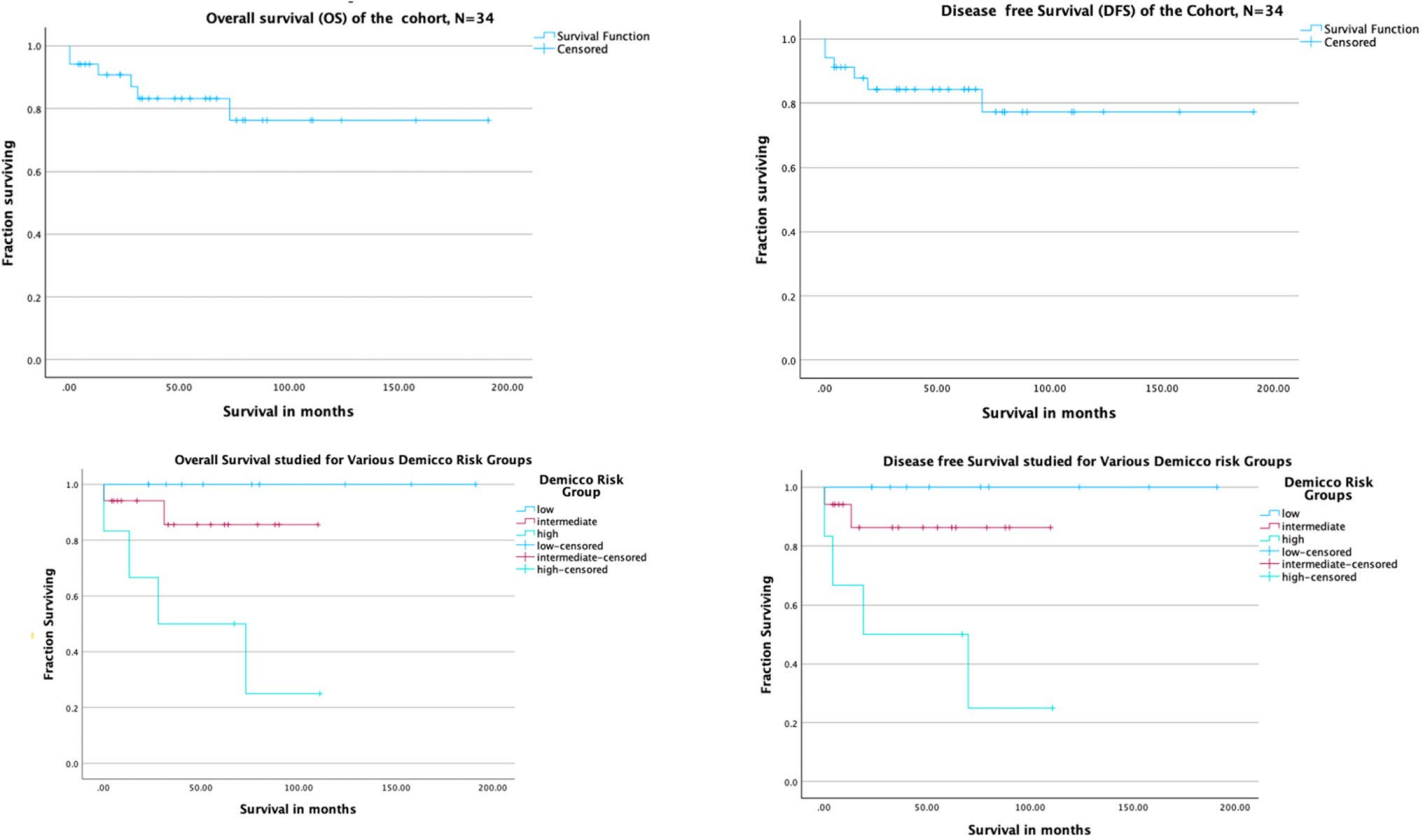
Kaplan–Meier curves for overall survival and disease-free survival of the cohort and Demicco risk groups

Median follow-up duration was 52 months (ranging from 6 to 192 months). The median DFS and OS had not reached for our cohort. The 5-year DFS was 77% (95% CI 126.5 to 180.8 months and standard deviation of 13.7 months). The 5-year OS was 82% (95% CI 126.6 to 180.9 months and standard deviation of 13.8 months). We saw four recurrences with disease-free interval (DFI) ranging from 13 to 73 months, with the lung being the site of metastases in all cases, without any local recurrence. Salvage treatment was attempted in three patients who had unresectable pulmonary recurrences, all managed with systemic chemotherapy with varying regimes including single agent adriamycin, docetaxel–cisplatin, and ifosfamide–adriamycin. All three had progressed while being on first line, and further salvage could only be attempted for one patient who was started on imatinib and supported with a single agent, ifosfamide. Median survival after recurrence was 9 months, and all patients who recurred died due to disease progression.

The 5-year DFS and OS in Demicco low-risk group were 100% and 100% respectively; intermediate-risk group 84% and 84%, respectively; and high-risk group 30% and 50% respectively (Fig. 1). None of our patients who were low risk had any recurrence. On univariate analysis for DFS, Demicco risk group and de Perrot staging were significant but none was significant in multivariate analysis (Table 3). On univariate analysis for OS, Demicco risk group and de Perrot staging were significant but both lost their predictive value in multivariate analysis (Table 4).

## Discussion

En bloc complete resection with negative margins remains the principle of the surgery for SFTs. Weiss et al. and Lorenz et al. recommended preoperative angiography for large tumors in the thoracic cavity, particularly when they were larger in volume, in which piecemeal excision may be anticipated [9, 21]. Lucarelli et al. studied the utility of TAE in tumors more than 10 cm in the largest dimensions, which is similar to our cohort of patients [3]. We observed that intraoperative blood loss was higher in comparison to non-angioembolized cases, but it is noteworthy that these two groups are not necessarily similar due to their difference in sizes/volumes and sites (Table 2). In our experience, we were able to use preoperative angioembolization to enable en bloc resection in all the patients in whom difficult resections were anticipated due to augmented tumor vasculature and larger volumes. We observed one-third of patients after angioembolization developing self-limiting fever, leukocytosis, and chest pain in the initial 48 h. The literature on post-angioembolization phenomena, including post-embolization syndrome, is scarce in the context of thoracic masses, unlike in liver tumors [22]. Further accidental embolization of spinal arteries is a major concern during TAE, which requires anticipating the risk, early identification, and management [23, 24]. In our cohort, one patient developed the same, which was successfully managed and did not have any further bearing on this patient’s disease management. Even with improvements in preoperative tools, including better imaging and growing experience in TAE, the surgical outcomes are not uniform due to the rarity of these tumors and different practices across different centers [25]. A meta-analysis by Mercer et al. had described the 30-day postoperative mortality of 0 to 3.6% and major morbidity of 16.7%, which is comparable to surgical outcomes mentioned in our study of 5.8% and 11.8%, respectively [25].

The role of adjuvant treatment in surgically treated SFT is not well defined, and only one patient in our study received the same as he was oligo-metastatic at presentation [25]. In a retrospective database review of over 600 SFTs, OS was similar for patients undergoing surgery alone compared to those receiving surgery and adjuvant radiation [26]. However, it is difficult to draw conclusions from such retrospective population-based studies, as they often include other types of sarcoma and may have a referral bias for adjuvant radiation, as these patients had larger tumors and incomplete/marginal resections. Adjuvant systemic treatment in the form of chemotherapy or targeted treatment for operated SFT is not established [25]. Risk stratification models like the Demicco model can help predict biological behavior and can help to prognosticate such patients [11, 17, 18, 27]. In our study, none of the patients with low risk in Demicco risk stratification had any recurrence, while 50% of high risk and 5.8% of intermediate risk had recurrences, similar to the risk predicted by the original study by Demicco et al. [11, 17]. However, in our cohort, neither the Demicco model nor the de Perrot staging yielded significant results in multivariate analysis for DFS and OS. This underlines the need to develop better prognostication models as more data matures and our understanding of the SFT sarcoma pathways evolves. The use of CT tumor volumetry as a preoperative surrogate for the Demicco model needs to be explored further, as observed in our study [17]. Long-term survival after surgery remains feasible and but close follow-up is necessary, as recurrences as late as 73 months have been noted in our cohort, highlighting the need for long-term surveillance. In our study, out of the four who had recurrence, one patient had recurrence after 5 years, which is similar to outcomes studied by Mercer et al. who reported a late recurrence after 5 years of 23.3% [25]. Recurrent diseases in our study were all systemic relapses, and salvage treatment with chemotherapy had dismal outcomes despite initial responses, which is similar to published literature [28, 29]. The role of targeted therapy, including further research into anti-angiogenic therapy, is an exciting field of research in solitary fibrous tumor management, with their current focus being as salvage treatment in recurred metastatic cases [30].

### Strength and Limitations of the Study

The major strength of the current study is that it is the single largest cohort of surgically treated SFTs from a tertiary cancer center in South Asia, discusses real-world outcomes, and reflects our current clinical practices. The limitations of the study include its retrospective nature, single-institution series, and relatively small numbers. However, through multi-institutional collaborative efforts through the National Cancer Grid of India, we hope to build a nationwide database for such rare tumors which may help in consensus building and develop better tools for the management of such diseases [31].

## Conclusion

Surgical resection with negative margins is the standard of care for thoracic solitary fibrous tumors. Preoperative angioembolisation can be beneficial in managing large tumors by reducing vascularity and facilitating en bloc resection. Patients in intermediate and high Demicco risk groups have a higher likelihood of late systemic recurrence, which is associated with poor prognosis. Therefore, long-term surveillance and the development of effective adjuvant and salvage therapies are crucial for improving outcomes in these high-risk patients.

## Data Availability

The data that support the findings of this study are available on reasonable request from the corresponding author.

## References

[1] Abu AW (2012) Solitary fibrous tumours of the pleura. Eur J Cardiothorac Surg 41:587–597 22345180 10.1093/ejcts/ezr009

[2] Piccinelli ML, Law K, Incesu RB, Tappero S, Cano Garcia C, Barletta F et al (2024) Demographic and clinical characteristics of malignant solitary fibrous tumors: a SEER database analysis. Cancers (Basel) 16(19):3331. 10.3390/cancers16193331 39409953 10.3390/cancers16193331 PMC11482613

[3] Lucarelli NM, Maggialetti N, Marulli G, Mariani P, Vilanova I, Mirabile A et al (2024) Preoperative embolization in the management of giant thoracic tumors: a case series. J Pers Med 14(10):1019. 10.3390/jpm14101019 39452527 10.3390/jpm14101019 PMC11508663

[4] Shi X, Liu X, Dong X, Wu H, Cai K (2022) Trends, symptoms, and outcomes of resectable giant mediastinal tumors. Front Oncol 12:820720. 10.3389/fonc.2022.820720 35186755 10.3389/fonc.2022.820720 PMC8854276

[5] Cardillo G, Facciolo F, Cavazzana A, Capece G, Gasparri R, Martelli M (2000) Localized (solitary) fibrous tumors of the pleura: an analysis of 55 patients. Ann Thorac Surg 70(6):1808–12. 10.1016/s0003-4975(00)01908-1 11156076 10.1016/s0003-4975(00)01908-1

[6] Harrison-Phipps KM, Nichols FC, Schleck CD, Deschamps C, Cassivi SD, Schipper PH et al (2009) Solitary fibrous tumors of the pleura: results of surgical treatment and long-term prognosis. J Thorac Cardiovasc Surg 138:19–25. 10.1016/j.jtcvs.2009.01.026 19577049 10.1016/j.jtcvs.2009.01.026 PMC2930758

[7] Luciano C, Francesco A, Giovanni V, Federica S, Cesare F (2010) CT signs, patterns, and differential diagnosis of solitary fibrous tumors of the pleura. J Thorac Dis 2(1):21–5. 10.3978/j.issn.2072-1439.2014.12.43. Erratum in: J Thorac Dis. 2014 Dec;6(12):E312 22263012 PMC3256431

[8] Fukuda I, Hizuka N, Ishikawa Y, Yasumoto K, Murakami Y, Sata A et al (2006) Clinical features of insulin-like growth factor-II producing non-islet-cell tumor hypoglycemia. Growth Horm IGF Res 16(4):211–6. 10.1016/j.ghir.2006.05.003 16860583 10.1016/j.ghir.2006.05.003

[9] Lorenz JM, Navuluri R (2019) Embolization of chest neoplasms: the next frontier in interventional oncology? Semin Intervent Radiol. 36(3):176–182. 10.1055/s-0039-1692658 31435125 10.1055/s-0039-1692658 PMC6699877

[10] England DM, Hochholzer L, McCarthy MJ (1989) Localized benign and malignant fibrous tumors of the pleura. A clinicopathologic review of 223 cases. Am J Surg Pathol 13:640. 10.1097/00000478-198908000-00003 2665534 10.1097/00000478-198908000-00003

[11] Demicco EG, Park MS, Araujo DM, Fox PS, Bassett RL, Pollock RE et al (2012) Solitary fibrous tumor: a clinicopathological study of 110 cases and proposed risk assessment model. Mod Pathol 25:1298–1306. 10.1038/modpathol.2012.83 22575866 10.1038/modpathol.2012.83

[12] Dacic S, Noguchi M, Tavora F, Demicco E, Calabrese F (2020) Solitary fibrous tumour of the thorax. In: WHO Classification of Tumours Editorial Board. Thoracic Tumours (5^th^ ed.). Lyon (France): International Agency for Research on Cancer. (WHO Classification of Tumours series, 5th edition; vol. 3)

[13] Santos RS, Haddad R, Lima CE, Liu YL, Misztal M, Ferrerira T et al (2005) Patterns of recurrence and long-term survival after curative resection of localized fibrous tumors of the pleura. Clin Lung Cancer 7:197–201. 10.3816/clc.2005.n.036 16354315 10.3816/clc.2005.n.036

[14] World Medical Association (2013) Declaration of Helsinki: ethical principles for medical research involving human subjects. JAMA 310(20):2191–2194. 10.1001/jama.2013.281053. hdl:10818/33790 24141714 10.1001/jama.2013.281053

[15] von Elm E, Altman DG, Egger M, Pocock SJ, Gøtzsche PC, Vandenbroucke JP (2014) The Strengthening the Reporting of Observational Studies in Epidemiology (STROBE) statement: guidelines for reporting observational studies. Int J Surg. 12:1495–1499 25046131 10.1016/j.ijsu.2014.07.013

[16] Gallastegui A, Cheung J, Southard T, Hume KR (2018) Volumetric and linear measurements of lung tumor burden from non-gated micro-CT imaging correlate with histological analysis in a genetically engineered mouse model of non-small cell lung cancer. Lab Anim 52(5):457–469. 10.1177/0023677218756457 29436921 10.1177/0023677218756457

[17] Demicco EG, Wagner MJ, Maki RG, Gupta V, Iofin I, Lazar AJ et al (2017) Risk assessment in solitary fibrous tumors: validation and refinement of a risk stratification model. Mod Pathol 30:1433–1442. 10.1038/modpathol.2017.54 28731041 10.1038/modpathol.2017.54

[18] de Perrot M, Fischer S, Bründler MA, Sekine Y, Keshavjee S (2002) Solitary fibrous tumors of the pleura. Ann Thorac Surg 74(1):285–293. 10.1016/s0003-4975(01)03374-4 12118790 10.1016/s0003-4975(01)03374-4

[19] IBM Corp. Released 2023. IBM SPSS statistics for Macintosh, Version 29.0.2.0 Armonk. IBM Corp, NY

[20] Flint A, Weiss SW (1995) CD-34 and keratin expression distinguishes solitary fibrous tumor (fibrous mesothelioma) of pleura from desmoplastic mesothelioma. Hum Pathol 26:428–31. 10.1016/0046-8177(95)90145-0 7535740 10.1016/0046-8177(95)90145-0

[21] Weiss B, Horton DA (2002) Preoperative embolization of a massive solitary fibrous tumor of the pleura. Ann Thorac Surg 73(3):983–985. 10.1016/s0003-4975(01)03117-4 11899221 10.1016/s0003-4975(01)03117-4

[22] Leung DA, Goin JE, Sickles C, Raskay BJ, Soulen MC (2001) Determinants of postembolization syndrome after hepatic chemoembolization. J Vasc Interv Radiol 12(3):321–326. 10.1016/s1051-0443(07)61911-3 11287509 10.1016/s1051-0443(07)61911-3

[23] Zhang Q, Li J, He G, Tang J, Zhang G (2023) Utility of intra-procedural cone-beam computed tomography imaging for the determination of the artery of Adamkiewicz suspected by angiography during transarterial embolization for hemoptysis. Diagn Interv Radiol 29:713–718. 10.4274/dir.2022.221646 36994610 10.4274/dir.2022.221646 PMC10679543

[24] Taterra D, Skinningsrud B, Pękala PA, Hsieh WC, Cirocchi R, Walocha JA et al (2019) Artery of Adamkiewicz: a meta-analysis of anatomical characteristics. Neuroradiology 61:869–880. 10.1007/s00234-019-02207-y 31030251 10.1007/s00234-019-02207-y PMC6620248

[25] Mercer R, Wigston C, Banka R, Cardillo G, Benamore R, Nicholson AG et al (2020) Management of solitary fibrous tumours of the pleura: a systematic review and meta-analysis. ERJ Open Res 6(3):00055–02020 32832532 10.1183/23120541.00055-2020 PMC7430150

[26] Haas RL, Walraven I, Lecointe-Artzner E, van Houdt WJ, Strauss D, Schrage Y et al (2020) Extrameningeal solitary fibrous tumors-surgery alone or surgery plus perioperative radiotherapy: a retrospective study from the global solitary fibrous tumor initiative in collaboration with the Sarcoma Patients EuroNet. Cancer 126(13):3002–3012. 10.1002/cncr.32911 32315454 10.1002/cncr.32911 PMC7318349

[27] Reisenauer JS, Mneimneh W, Jenkins S, Mansfield AS, Aubry MC, Fritchie KJ et al (2018) Comparison of risk stratification models to predict recurrence and survival in pleuropulmonary solitary fibrous tumor. J Thorac Oncol 13(9):1349–1362. 10.1016/j.jtho.2018.05.040 29935303 10.1016/j.jtho.2018.05.040

[28] O’Neill AC, Tirumani SH, Do WS, Keraliya AR, Hornick JL, Shinagare AB et al (2017) Metastatic patterns of solitary fibrous tumors: a single-institution experience. AJR Am J Roentgenol 208:2–9. 10.2214/AJR.16.16662 27762594 10.2214/AJR.16.16662

[29] Baldi GG, Stacchiotti S, Mauro V, Dei Tos AP, Gronchi A, Pastorino U et al (2013) Solitary fibrous tumor of all sites: outcome of late recurrences in 14 patients. Clin Sarcoma Res 3:4. 10.1186/2045-3329-3-4 23551825 10.1186/2045-3329-3-4 PMC3637255

[30] Zhang R, Yang Y, Hu C, Huang M, Cen W, Ling D et al (2023) Comprehensive analysis reveals potential therapeutic targets and an integrated risk stratification model for solitary fibrous tumors. Nat Commun 14:7479. 10.1038/s41467-023-43249-4 37980418 10.1038/s41467-023-43249-4 PMC10657378

[31] Pramesh CS, Badwe RA, Sinha RK (2014) The national cancer grid of India. Indian J Med Paediatr Oncol 35(3):226–227. 10.4103/0971-5851.142040 25336795 10.4103/0971-5851.142040 PMC4202620

